# Genetic Testing Protocol Reduces Costs and Increases Rate of Genetic Diagnosis in Infants with Congenital Heart Disease

**DOI:** 10.1007/s00246-017-1685-7

**Published:** 2017-07-19

**Authors:** Gabrielle C. Geddes, Donald Basel, Peter Frommelt, Aaron Kinney, Michael Earing

**Affiliations:** 1 Department of Pediatrics, Medical College of Wisconsin, Milwaukee, WI USA; 2 Herma Heart Center, Children’s Hospital of Wisconsin, 9000 West Wisconsin Ave, MS #716, Milwaukee, WI 53226 USA

**Keywords:** Genetic testing, Congenital heart disease, Microarray, 22q11.2 Deletion syndrome

## Abstract

Genetic testing is routinely performed on infants with critical congenital heart disease (CHD). This project reviewed the effect of implementing a genetic testing protocol in this population. Charts of infants with critical CHD were reviewed for genetic testing and results across two time periods: the time before implementation of a genetic testing protocol (pre-protocol) and the time after implementation (post-protocol). The use of karyotype, 22q11.2 Deletion testing, and chromosomal microarray were compared across these two time periods. Records of 891 infants were reviewed. 562 (63%) had at least one of the target genetic tests completed. During the pre-protocol time period, 66% of patients who had genetic testing underwent multiple tests versus 24% during the post-protocol time period (*p* < 0.01). The rate of patients who underwent genetic testing increased from 60% in the pre-protocol time period to 77% in the post-protocol time period (*p* < 0.01). The rate of diagnosis of genetic conditions during the pre-protocol period was 26% versus 36% during the post-protocol period (*p* = 0.01). There was a reduction in cost to patients by $5105.59 per diagnosis during the post-protocol period. Patients with critical CHD in the post-protocol period were less likely to undergo multiple genetic tests and more likely to have a diagnosis of genetic disease. In addition there was a significant reduction in cost per diagnosis during the post-protocol time period. Genetic testing protocols for infants with critical CHD promoted more efficient use of genetic testing and increased the rate of diagnosis of genetic conditions in this population.

## Introduction

Patients with critical congenital heart disease (CHD) have a high rate of copy number variants, particularly 22q11.2 Deletion Syndrome, yet clinical use of chromosomal microarray (CMA) in patients with CHD is unpredictable and not standardized [1–5]. Previous work has demonstrated copy number variants demonstrated by CMA in 18–31% of patients with CHD [1, 3, 6–8]. These studies differ on inclusion of syndromic versus isolated CHD, critical CHD versus all CHD, as well as description of CHD. Clinically relevant recommendations for testing and evaluation are available, but have yet to be fully embraced [9]. Previous assessments of rate of genetic diagnosis following genetic evaluation in infants with critical CHD were 25% [10]. This quality improvement project was focused on increasing the consistency of genetic testing in infants with critical CHD and looking at the effects a genetic testing protocol had on genetic testing utilization and diagnosis rate.

## Materials and Methods

### Study Overview

In order to determine the rates of genetic testing in infants with critical CHD the genetic testing practices and yields for two time periods were examined. Baseline data were obtained through a project approved by the Institutional Review Board which resulted in the genetic testing protocol. Follow-up data were obtained as part of an Institutional Review Board exempt status quality improvement project determining the adherence to the protocol.

### Patient Population

The Society for Thoracic Surgeons Database was queried for infants who had CHD requiring surgical intervention at less than a year of age. The first time period, or “pre-protocol” time period, includes infants who underwent cardiac surgery at less than one year of age on or after January 1, 2010 who were born on or before December 31, 2014. The second time period, or “post-protocol” time period, includes infants born between January 1, 2015 and June 30, 2016 who had cardiac surgery at less than a year of age before June 30, 2016. Infants born at less than 30 weeks gestation or who underwent patent ductus arteriosus repair were excluded.

### Genetic Testing

All genetic testing recorded in the medical record was identified and was marked as normal or abnormal based on how it was clinically reported. Focus was on the three most commonly utilized genetic tests addressed by the protocol: G-Banded Karyotype, diagnostic testing for 22q11.2 deletion, and chromosomal microarray (CMA). The G-banded Karyotype has a resolution of identifying chromosomal abnormalities greater than five megabases. Karyotype testing can detect balanced translocations and structural changes not resulting in a change in copy number that could be missed by microarray and can detect low level chromosomal mosaicism. The method for diagnostic testing for 22q11.2 deletion included florescence in situ hybridization (FISH) for TUPLE1 and quantitative polymerase chain reaction (QPCR) for TBX1. FISH probe for TUPLE1 has a sensitivity of greater than 95% for 22q11.2 deletion syndrome and was available for the duration of the study. QPCR for TBX1 copy number became available to infants at Children’s Hospital of Wisconsin in August 2011 and has a sensitivity of greater than 95% for 22q11.2 deletion syndrome. In September 2012 this assay began to include copy number of CRKL to further enhance the sensitivity of detecting 22q11.2 deletions. The CMA utilized at Children’s Hospital of Wisconsin reports deletions larger than 200 kilobases and duplications larger than 500 kilobases. CMA is the most sensitive genetic test out of these options, and will pick up everything detectable by karyotype and 22q11.2 deletion testing with the exclusion of balanced translocations and low level chromosomal mosaicism which may only be detectable by karyotype.

### Protocol Implementation

The genetic testing protocol was implemented on January 1, 2015. The protocol was introduced to the department of pediatrics and affected clinical divisions (cardiac intensive care unit, cardiology, cardiothoracic surgery, genetics, and neonatal intensive care unit) through departmental lectures and division meetings starting in April 2014. Our basic testing protocol is demonstrated in Table 1.

**Table 1:** Genetic testing protocol, *CHD* congenital heart disease

Patient features	What to order
*CHD with features suggestive of trisomy 21 or Turner syndrome*	Karyotype
	*Contact genetics for genetic counseling*
*CHD with features of trisomy 13 or trisomy 18*	STAT FISH for 13, 18, 21, X and Y
	*Consult genetics for further testing*
*Conotruncal congenital heart lesion*	
Interrupted aortic arch	22q11.2 deletion testing chromosomal microarray
Pulmonary atresia with ventricular septal defect	
Tetralogy of fallot	
Truncus arteriosus	
Malaligned ventricular septal defect and/or features typical of 22q11.2 deletion syndrome	
*Heterotaxy*	Chromosomal microarray heterotaxy panel
*CHD without features of the above categories*	Chromosomal microarray

### Diagnosis Rate

All genetic diagnoses listed in the medical record were noted (e.g., Hemophilia, Sickle Cell Disease). Genetic diagnoses associated with congenital heart disease (e.g., CHARGE Syndrome, Trisomy 21) were used to classify patients as being diagnosed with a genetic condition. For the specific protocol diagnosis rate the genetic diagnosis was only recorded if it was a direct result of the three target tests ordered. If a patient had a genetic diagnosis not made by the testing it was not recorded (e.g., a patient with Trisomy 21 underwent screening for 22q11.2 deletion only or if a patient with CHARGE Syndrome had a normal microarray) and this protocol diagnosis rate was utilized to determine the calculation of cost per diagnosis. All charts were reviewed for genetic diagnosis by a single author for consistency (GCG).

### Value and Cost Analysis

In order to determine the effect of the protocol on cost to patients we assessed cost and compared pre-protocol to post-protocol periods. To determine cost baselines we queried three large genetic testing providers on the same day and averaged the quoted prices of the three providers and averaged them to create a cost for each test. The average costs at a single discrete time point were utilized to standardize for changes in cost over time. These were $895.17 for karyotype, $669.92 for 22q11.2 Deletion Testing, and $2615.33 for CMA. To determine the effect on value we used the cost data per patient and per diagnosis.

### Data Analysis

Descriptive statistics identified the proportion of specific CHD group for each classification system represented within the sample and the frequency of abnormal genetic test results in proportion to tests completed. Two-tiered Fisher’s exact test was utilized to determine statistical significance of changes between groups.

## Results

### Population Description

Medical records of 891 infants were examined. There were 509 (57%) male infants and 382 (43%) female infants. Among these 891 infants 109 were diagnosed with Trisomy 21 and 36 were diagnosed with 22q11.2 Deletion Syndrome. Overall 562 (63%) had at least one of the three genetic tests completed. Population characteristics are summarized in Table 2 by time period.

**Table 2:** Patient characteristics by time period

	Pre-protocol	Post-protocol
Total patients	733	158
Male	421 (57%)	88 (43%)
Female	312 (43%)	70 (44%)
Trisomy 21	87 (12%)	22 (14%)
22q11.2 deletion syndrome	28 (4%)	8 (5%)
Tested with one of 3 target tests	441 (60%)	121 (77%)
Karyotype	307 (42%)	33 (21%)
22q11.2 deletion testing	243 (33%)	23 (15%)
Microarray	292 (40%)	94 (59%)
Untested patients	292 (40%)	37 (23%)

### Testing Practices

**Fig. 1:**
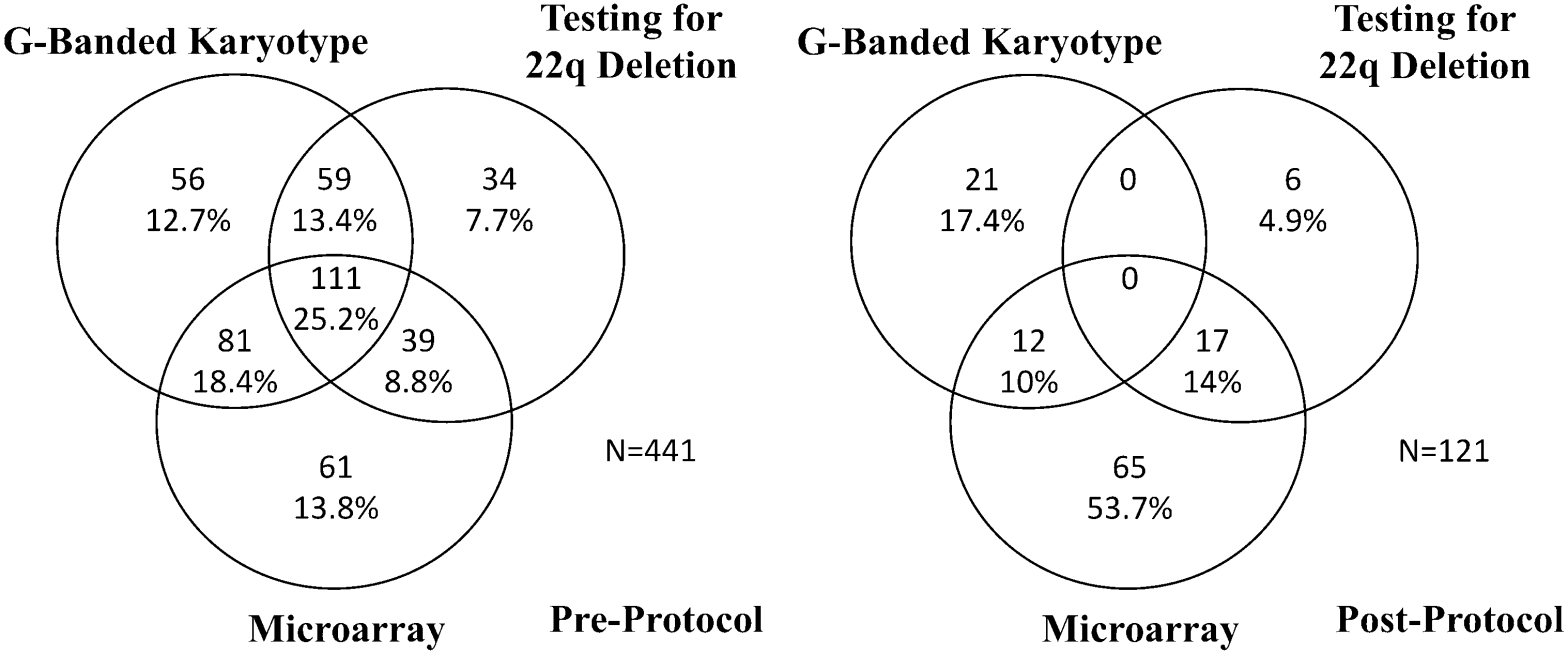
Genetic testing patterns by Time Period. Pattern of using multiple genetic testing pre-protocol (*left*) and post-protocol (*right*) demonstrating reduction in frequency of undergoing multiple genetic tests at once

During the pre-protocol time period 66% of patients who had genetic testing underwent multiple tests. During the post-protocol time period 24% of patients who had genetic testing underwent multiple tests. This was found to be a significant decrease (*p* < 0.01) in utilization of multiple genetic tests. Testing patterns across the two time periods are demonstrated in Fig. 1.

During the pre-protocol time period 60% of patients had genetic testing. During the post-protocol time period 77% of patient had genetic testing. This was found to be a significant increase (*p* < 0.01) of patients undergoing genetic testing in the post-protocol period.

### Yield of Testing and Diagnosis Rate

In the pre-protocol time period the rate of abnormal karyotypes was 18%, the rate of abnormal 22q11.2 deletion testing was 9%, and the rate of abnormal microarray was 24%. In the post-protocol time period the rate of abnormal karyotype was 76%, the rate of abnormal 22q11.2 deletion testing was 26%, and the rate of abnormal microarray was 22%. The increase in yield of karyotype was found to be significant (*p* < 0.01) as was the increase in yield of 22q11.2 deletion testing (*p* = 0.03). There was no difference in yield of microarray between the pre-protocol and post-protocol time periods (*p* = 0.78). This is illustrated in Table 3. There were no results detected by karyotype or 22q11.2 deletion testing in this cohort that was not detectable with microarray in this patient population.

**Table 3:** Yield of genetic testing by time period

	Pre-protocol time period			Post-protocol time period		
	Completed	Abnormal	Abnormal (%)	Completed	Abnormal	Abnormal (%)
Karyotype	307	56	18	33	25	76
22q11.2 testing	243	23	9	23	6	26
Microarray	292	70	24	94	21	22

The pre-protocol time period had increased utilization of Karyotype and 22q11.2 testing with low yield, but in the post-protocol period these tests were used more efficiently as reflected by an increased yield in abnormal test results. The rate of abnormalities identified by microarray showed no significant difference between the pre-protocol and post-protocol period showing that expanded utilization increases number of patients identified with chromosomal anomalies

The breakdown of testing patterns and yield of testing by lesion is demonstrated in Table 4 which classifies cardiac lesions based on the National Birth Defect Prevention Study classification system [10]. Results of this breakdown are as anticipated. Trisomy 21 is more likely to be found in patients with atrioventricular septal defects making the karyotype more likely to be abnormal in patients with these lesions. Patients with 22q11.2 Deletion Syndrome are more likely to have conotruncal lesions and 22q11.2 deletion testing is more likely to be abnormal in this group. Of note, there was a higher yield for microarray testing in conotruncal lesions than 22q11.2 deletion testing alone, likely reflecting the increased clinical utility of microarray in detecting genetic disorders. There was no significant difference in yield of microarray across lesion types with a single exception. Patient with septal lesions who had microarray were significantly more likely to have an abnormal microarray (*p* = 0.0005). We suspect this is a reflection of clinical ascertainment bias given that only 29% patients with septal lesions had microarray testing completed.

**Table 4:** Yield of testing by cardiac lesion

Lesion	*n*	Karyotype	Abn	22q	Abn	CMA	Abn
APVR	29	1	0	0	0	10	0
AVSD	97	39	33 (87%)	1	0	12	3 (25%)
Complex	60	25	5 (25%)	21	0	29	6 (24%)
Conotruncal	277	117	17 (14.5%)	139	27 (19%)	132	31 (23%)
Heterotaxy	52	28	0	21	0	38	6 (16%)
LVOTO	195	77	11 (14%)	50	1 (2%)	92	21 (23%)
RVOTO	53	20	2 (10%)	20	0	32	8 (25%)
Septal	93	22	12 (55%)	9	1 (11%)	27	13 (48%)
Other	35	11	1 (9%)	5	0	14	3 (21%)
Total	891	340	81 (24%)	266	29 (11%)	386	91 (24%)

This table demonstrates the yield of testing by National Birth Defect Prevention Study cardiac lesion classification. Karyotypes were most often abnormal in the AVSD group, followed by the septal group. 22q testing was abnormal most commonly in patients with conotruncal lesions. There were no statistically significant differences in CMA yield based on cardiac lesion with one exception. Patients with septal lesions were significantly more likely (*p* = 0.0005) to have an abnormal microarray when compared to other groups

*Abn* abnormal, *APVR* anomalous pulmonary venous return, *AVSD* atrioventricular septal defect, *CMA* chromosomal microarray, *LVOTO* left ventricular outflow tract obstruction, *RVOTO* right ventricular outflow tract obstruction, 22q = 22q11.2 deletion Testing

The rate of diagnosis of genetic conditions associated with congenital heart disease during the pre-protocol period was 26%. The rate of diagnosis of genetic conditions associated with congenital heart disease during the post-protocol period was 36%. This increase in diagnosis rate was significant (*p* = 0.01). When patients with Trisomy 21 were excluded the rate of diagnosis of genetic conditions during the pre-protocol period was 16% and during the post-protocol period was 26%. This increase in diagnosis rate among patients without Trisomy 21 was also significant (*p* = 0.01).

### Cost of Testing

Prior to the protocol initiation, fewer patients underwent genetic testing (60 vs. 77%), but of the patients who did undergo genetic testing, they underwent multiple tests more frequently (66 vs. 24%, *p* < 0.01). As result, the average cost per patient for genetic testing was higher prior to the initiation of the protocol, $2720.77 verse $2403.27. When we analyzed the cost per genetic diagnosis from testing ordered we found similar results with the average cost per genetic diagnosis being significant less after the protocol was initiated, $11,427.24 verse $6321.65. The cost of testing per time period is further demonstrated in Table 5.

**Table 5:** Cost of genetic testing by time period

Testing combination	Pre-protocol time period			Post-protocol time period		
	*n*	Diagnosis from Testing	Cost	*n*	Diagnosis from Testing	Cost
Karyotype + 22q + CMA	111	18	$464,048.82	0	0	0
Karyotype + 22q	59	4	$92,340.31	0	0	0
Karyotype + CMA	81	24	$284,391.00	12	6	$42,132.00
QPCR + CMA	39	9	$128,124.75	17	3	$55,849.25
Karyotype only	56	33	$50,129.52	21	21	$18,798.57
22q only	34	2	$21,290.18	6	4	$4,019.52
CMA only	61	15	$159,535.13	65	12	$169,996.45
Patients tested	441	105	$1,199,859.71	121	46	$290,795.79
Patients untested	292	0	$0	37	0	$0
Total	733	105	$1,199,859.71	158	46	$290,795.79
**Average Cost per Tested Patients**	**$2720.77**			**$2403.27**		
**Average Cost per Diagnosis**	**$11,427.24**			**$6321.65**		

This table demonstrates the pre-protocol testing patterns and associated cost to the patient in addition to the number of genetic diagnoses made by the test specified. The bolded number reflect total average cost per tested patient and total cost per diagnosis for the two time periods. This demonstrates a post-protocol reduction in average cost per patient who underwent genetic testing by $317.50 and a post-protocol reduction on average cost per diagnosis by $5105.59. *CMA* Chromosomal Microarray, 22q = 22q11.2 deletion testing

## Discussion

Our data demonstrate the utility of thoughtful genetic testing protocols in infants with critical CHD. The protocol significantly increased the rate of diagnosis of genetic conditions. A significant concern in implementing this protocol was that the yield of CMA would go down with expanded testing as appropriate individuals were undergoing testing and there was selection bias in the pre-protocol population. Our data demonstrate that the yield of microarray is consistent in this population and expanded testing resulted in an increased number of children identified to have chromosomal anomalies. Our diagnosis rate of genetic conditions associated with congenital heart disease is significantly higher than what has been published. The most recent assay of infants with critical congenital heart disease who underwent genetic assessment reported a diagnosis rate of 25% [11]. One potential influence on our data is that a dedicated clinical cardiovascular genetics program was started 6 months into the post-protocol period and the increased participation of geneticists in the care of this population. The diagnosis rate for this new program has been high compared to published rates at 39% (38/97) for infants with CHD assessed less than one year of age without Trisomy 21. This likely synergistically affected the diagnosis rate for this population of diagnoses not detected with CMA.

Importantly, our data suggest that the protocol reduced the overall cost of testing for patients. While the protocol resulted in more patients undergoing genetic testing, it reduced the number of patients undergoing multiple tests at once, which reduced the overall cost per patient by $317.50. Costs and charges are complex and can vary significantly between institutions. There was sincere concern that implementing this protocol could increase costs to patients and families. Our data suggest that a genetic testing protocol has the opposite effect. The cost per diagnosis was reduced significantly by $5105.59. This reflects that in addition to patients undergoing fewer redundant tests at once, more patients were undergoing more appropriate testing. More appropriate use of genetic testing is further evident by the statistically significant increase in yield for karyotype (18–76%) and 22q11.2 deletion testing (9–26%) after initiation of the protocol.

There are still clinical nuances to choosing genetic testing this protocol does not full address. Microarray testing has increased resolution to detect clinically significant chromosomal anomalies compared to karyotype, but important limitations of microarray include the inability to detect balanced translocations or determine the structural location of an anomaly as well as the inability to detect low level chromosomal mosaicism, which can be important when considering mosaic conditions like Turner Syndrome. Microarray is also not always the best test for the situation. For example, testing with karyotype Trisomy 21 is still appropriate as karyotype is the recommended standard of care to assess for translocations [12]. All of these genetic tests still have clinical roles, but our data suggest that a protocol helps patients with critical congenital heart disease undergo more appropriate clinical application of these tests.

Our study does have limitations. We are a tertiary referral center, and as a result it is possible that additional testing following discharge was performed at an outside institution that was not available at the time of chart review. As all charts were reviewed for testing by a single author (GCG) with a consistent, standard method it is likely limitation would be uniform throughout each time period. In addition, cost and value calculations are highly variable and continue to evolve over time, making retrospective reviews challenging.

Despite these limitations, we feel that our data support a standardized genetic testing protocol for infants with critical cardiac disease can significantly impact the rate of identification of chromosomal anomalies and is cost effective in this complex, high risk population. We recommend institutions consider implementing genetic testing protocols for this population, ideally in conjunction with a medical geneticist or genetic counselor.
